# Antithymocyte Globulin as Second-Line Therapy in Graves Orbitopathy—Preliminary Results From a Prospective Single-Center Study

**DOI:** 10.3389/fendo.2022.871009

**Published:** 2022-05-09

**Authors:** Monika Sarnat-Kucharczyk, Maria Świerkot, Gabriela Handzlik, Grażyna Kulawik, Krystyna Jagoda, Iga Grochoła-Małecka, Joanna Fryżewska, Ewa Mrukwa-Kominek, Jerzy Chudek

**Affiliations:** ^1^ Department of Ophthalmology, Faculty of Medical Sciences in Katowice, Medical University of Silesia, Katowice, Poland; ^2^ Department of Internal Medicine and Oncological Chemotherapy, Faculty of Medical Sciences in Katowice, Medical University of Silesia, Katowice, Poland; ^3^ Department of Hematology and Bone Marrow Transplantation, Faculty of Medical Sciences in Katowice, Medical University of Silesia, Katowice, Poland

**Keywords:** Graves’ orbitopathy, Graves’ disease, antithymocyte globulin, proptosis, thyroid eye disease

## Abstract

**Objective:**

Management of Graves’ orbitopathy remains a challenge. Our previous case report has shown promising results for rabbit antithymocyte globulin (rATG) in the treatment of Graves’ orbitopathy.

**Design:**

We present the response of 7 individuals with active moderate-to-severe steroid-resistant Graves’ orbitopathy to rATG, representing preliminary results from a prospective single-center study.

**Methods:**

rATG was administered intravenously at a dose of 0.8–1.0 mg/kg daily (cumulative dose of 150–200 mg). The primary outcome measures at weeks 24 and 48 were ≥2-point reduction in Clinical Activity Score from baseline, a proptosis response, a diplopia response, and improvement of distant best-corrected visual acuity and mean retinal sensitivity. Key secondary outcomes included stabilization of ganglion cell complex thickness, a decrease of retinal nerve fiber layer in OCT, and a reduction in CD4/CD8 ratio and TRAb at 48 weeks.

**Results:**

An improvement in clinical activity score was observed in all patients, with disease inactivation in 3 cases. Proptosis reduction equal to or greater than 2 mm was noted for 8 of 10 eyes. Diplopia improved in three of 6 patients. There was an improvement in best-corrected visual acuity (from 0.69 to 0.78) and mean retinal sensitivity (from 20.8 to 23.5 dB). In addition, there was a long-lasting improvement in CD4/CD8 ratio in 6 patients. Two patients experienced adverse events (influenza and serum sickness).

**Conclusion:**

rATG therapy offers a long-lasting improvement in moderate-to-severe steroid-resistant Graves’ orbitopathy with improvement in functional vision (reduction of diplopia, improvement of visual acuity, retinal sensitivity, and VEP pattern). The therapy is well-tolerated.

**Clinical Trial Registration:**

ClinicalTrials.gov, identifier NCT05199103.

## 1 Introduction

Graves’ orbitopathy (GO), also known as thyroid eye disease, is the most common extrathyroidal manifestation of Graves’ disease, affecting 25% of patients with this complex autoimmune disorder (1). Graves’ disease is more prevalent in women than in men; however, if present in men, it takes a more severe form. Smoking and radioiodine therapy are strong risk factors for the development of sight-threatening orbitopathy (2).

Most cases of GO are benign, but 5%–6% of patients are classified as an active moderate-to-severe disease, in whom corticosteroid systemic administration is a mainstay of treatment recommended by EUGOGO (3). However, 20%–30% of patients refractory to standard steroid therapy require second-line treatment with repeated and high courses of corticosteroids, orbital radiotherapy, cyclosporin A, or rituximab. Over the past 5 years, novel hypotheses in the pathogenesis of GO created potential new therapeutic targets affecting immunological and inflammatory reactions, survival and proliferation of fibroblasts and adipocytes, and extracellular matrix production (4, 5). Among emerging agents are monoclonal antibodies against the interleukin-6 receptor (tocilizumab) and the insulin-like growth factor 1 receptor (teprotumumab) (5).

The mechanisms underlying the development of GO are complex and still not entirely elucidated, with key pathogenetic processes applying to autoimmune-induced extraocular muscle enlargement, orbital fat expansion, fibroblasts proliferation followed by their differentiation into myofibroblasts and adipocytes (6). Early orbital T-cell infiltration and local release of proinflammatory cytokines emerge as a triggering mechanism and become a basis for further therapeutic research. Infiltration of CD4+ cells to orbital tissues plays an essential role in the molecular pathways leading to the proliferation and differentiation of orbital fibroblasts. T regulatory (Treg) cells are considered substantial for suppression of immunological responses in autoimmune disorders. The activity of Treg cells may be influenced by the abnormal CD4 to CD8 ratio observed in GO (7).

Rabbit antithymocyte globulin (rATG, Thymoglobulin) is an anti-T-cell agent widely used in the management of acute cellular rejection after organ transplantation. rATG induces T-cell depletion and affects the cell surface and adhesion molecules regulating T-cell function. It has also anti-B-cell activity *via* induction of naïve plasma B-cell apoptosis (8). *In vitro*, incubation of peripheral blood mononuclear cells from GO patients with rATG resulted in restored CD4 to CD8 ratio and significantly increased the number of T CD3+ lymphocytes with the phenotype of Treg cells: FOXP3+ CD4+ CD25+ CD127^low^ (7). Of note, Lee et al. described the resolution of GO shortly after induction therapy with ATG (1.5 mg/kg/dose for five doses) with a triple immunosuppressive regimen (including glucocorticoids) in the kidney transplantation recipient previously untreated for GO. The clinical improvement, in this case, was followed by the disappearance of TSHR antibody titters that remained undetectable 12 months after the procedure (9).

Compressive optic neuropathy in the course of GO causes damage to the retinal ganglion cell axons of the optic nerve by mechanical damage. It usually results in a progressive and structural and functional deterioration of the optic nerve (10). The evaluation of both structure and function in optic neuropathies at the time of presentation provides crucial information about the status of the disease and is useful for monitoring the clinical progression and recovery after treatment (11).

It was shown that the peripapillary retinal nerve fiber layer (RNFL) is altered in compressive optic neuropathies of different causes depending on the stage of the disease (12).

In this paper, we report preliminary analyses of the ongoing single-center academic-driven research on the efficacy and safety of rATG in patients with steroid-resistant moderate-to-severe Graves’ orbitopathy.

## 2 Patients and Methods

This prospective interventional, single-center study enrolled adult patients with active moderate-to-severe GO after ineffective treatment with moderate-to-high doses of glucocorticoids. The study protocol was approved by the local bioethics committee of the Medical University of Silesia (KNW-1-075/N/8/K). All patients gave their written informed consent.

Euthyroid adult patients (>18 years of age) with active moderate-to-severe GO, with clinical activity score (CAS) of ≥3/7, and who were previously ineffectively treated (partial response, recurrence, or progression of symptoms) with moderate-to-high doses of glucocorticoids (at least 4.5 g of methylprednisolone) were considered eligible for enrollment.

The exclusion criteria were as follows: hypersensitivity to rabbit proteins or any product excipients, active acute or chronic infections, latent tuberculosis, leucopenia below 3,000/μl, lymphopenia below 400/μl, thrombocytopenia below 75,000/μl, coagulation disorders, active malignancy, and pregnancy.

Presented results describe effectiveness in seven patients who met all the inclusion criteria. Each eligible patient received 0.8–1.0 mg/kg of rATG (cumulative dose of 150–200 mg given intravenously in two or three divided doses, 24 h apart) after premedication with 1 mg of clemastine—and an antihistaminic agent orally, methylprednisolone (in the total dose of 375 mg), and 1,000 mg paracetamol given intravenously. Subsequent doses were preceded by complete blood count assessment (CBC) and dose postponement in case of lymphocyte count <100/μl (required in a single case).

To assess the efficacy and safety of rATG therapy, patients were evaluated at baseline and 6, 12, 24, and 48 weeks. Baseline evaluation involved medical history, physical examination, including eye examination, laboratory assessment thyroid-stimulating hormone (TSH), flow cytometry, TSH-receptor antibody (TRAb) titer, CBC), and orbital magnetic resonance imaging (MRI). Physical re-evaluations were conducted by the same endocrinologist and ophthalmologist.

Eye examinations were performed using:

- Hertl’s ophthalmometry,
- seven-point CAS assessment (spontaneous retrobulbar pain, pain on attempted eye movements, conjunctival hyperemia, eyelid redness, chemosis, swelling of the caruncle, swelling of the eyelids),
- classification of Graves’ orbitopathy: NOSPECS (0, no signs or symptoms; 1, only signs no symptoms; 2, soft-tissue involvement; 3, proptosis [amount of protrusion of the eye from the orbital rim]; 4, extraocular muscle involvement; 5, corneal involvement; 6, sight loss),
- diplopia Gorman’s scale: a score of 0 indicates no diplopia; 1, intermittent diplopia; 2, inconstant diplopia, and 3, constant diplopia,
- Ishihara color plates,
- distant uncorrected/best-corrected visual acuity (DUCVA/DBCVA),
- static visual field—mean sensitivity (MS)
- pattern visual evoked potentials (PVEP),
- posterior segment optical coherent tomography (OCT) of ganglion cell complex (GCC) in the macula and the retinal nerve fiber layer (RNFL) around the optic nerve.

### 2.1 Visual Evoked Potential Measurement

Visual evoked potential (VEP) measurements were performed using the Reti-Port electrophysiological apparatus from Roland Consult (Brandenburg a.d. Havel, Germany), following the standards of the International Society for Clinical Electrophysiology of Vision (ISCEV) (13). A black and white checkerboard pattern with alternating phase change (pattern reversal VEP, PVEP) was used to elicit a visual cortical response. The reversal rate was 2 reversals/s. Each VEP test was preceded by checking the visual acuity of the distance in the patient’s correction with glasses using Snellen charts, an intraocular pressure test using a Goldmann applanation tonometer. Before the fundus examination, short-acting (4–6 h) mydriatics were given: parasympatholytic (1% Tropicamidum, Polfa, Warsaw, Poland) or sympathomimetic (10% Neosynephrin-POS, Ursapharm, Saarbrücken, Germany) drops. The amplitude and the latency of the P100 (PVEP) waves were analyzed.

### 2.2 Flow Cytometry

Flow cytometry was performed on the lymphocyte subpopulation extracted from peripheral blood samples collected by venipuncture to EDTA tubes. The cells were stained using a reagent kit with six-color fluorochrome-labeled monoclonal antibodies (BD Multitest 6-color TBNK, Becton Dickinson Biosciences): CD3-FITC, CD16-PE + CD56-PE, CD45-PerCP-Cy5.5, CD4-PE-Cy7, CD19-APC, and CD8-APC-Cy7. The staining procedure was performed according to the manufacturer’s recommendations. Briefly, 50 μl of anticoagulated whole blood was added to 20 μl of reagent and incubated for 15 min in the dark at room temperature. Erythrocytes were briefly lysed, and samples were immediately acquired in an 8-color flow cytometer (FACSCanto II, Becton Dickinson Biosciences). In addition, the WBC count and the percentage of lymphocytes among leucocytes were counted on the Sysmex XN-10 cell counter. The acquisition process was followed by analysis according to the manufacturer’s instructions. The results were expressed as the percentage of T, T helper, T suppressor, B, and NK cell subpopulations among the lymphocytes and as the absolute number of these cells per 1 µl of blood. For further analysis, CD4/CD8 ratio was calculated.

### 2.3 Measurement of Clinical Outcomes

The primary outcome measures at weeks 24 and 48 were as follows: ≥2-point reduction in CAS from baseline, a proptosis response (defined as ≥2 mm reduction in proptosis), a diplopia response (defined as a reduction in diplopia of ≥1 grade from baseline using Gorman’s subjective diplopia score), and improvement of distant best-corrected visual acuity (considered significant if improved by ≥1 row in Snellen visual acuity chart) and mean retinal sensitivity (considered significant if increased by ≥2 dB).

Key secondary outcomes included: stabilization of ganglion cell complex thickness (defined as a change of less than ±2 μm), a decrease of retinal nerve fiber layer in OCT ≥2 μm, increase in 1° amplitude in Pattern VEP by 1 µV, changes in CD4/CD8 ratio (considered significant if reduced by at least 50%), and changes in TRAb.

### 2.4 Statistical Analysis

Data are presented as median values with interquartile ranges (1-3Q). Mean values were calculated to show changes in quantitative data with time. ANOVA for repeated measurements with Dunnett’s test as *post hoc* test and Student’s *t*-test for dependent data were conducted, as appropriate. Statistical significance was set at a *p*-value below 0.05.

## 3 Results

### 3.1 Study Group Characteristics

Seven patients (5 women and 2 men) with steroid-resistant and active Graves’ orbitopathy (CAS ≥4 pts) have completed the study protocol and were included in the intention-to-treat analysis. There were no dropouts, and all patients were examined after 6, 12, 24, and 48 weeks after the administration of rATG. Demographic data are presented in **Table 1**. Ophthalmologic examination and analysis included fourteen eyes.

**Table 1:** Baseline patients’ clinical data.

Sex [*N* (%)]	
Men	2 (29)
Women	5 (71)
Age (years)	59 (49–71)
Duration of Graves’ disease (years)	6 (4–15)
Duration of Graves’ orbitopathy (months)	11 (6–18)
Smokers (*n*) (%)	5 (71%)
Therapy of Graves’ disease [*N* (%)]	
Thiamazole	2 (29)
Thyroxine	5 (71)
Thyroidectomy	1 (14)
Radioactive iodine therapy	4 (57)
Patients with diplopia [*N* (%)]	6 (86)
CAS (pts)	4 (4–5)
TSH (uIU/ml)	0.54 (0.42–1.33)
FT4 (ng/dl)	1.01 (0.91–1.18)
Anti-TSH receptor (TRAb) (IU/ml)	2.08 (1.26–38.3)

Data are shown as medians with interquartile range.

Severe violations of the study protocol took place in two patients (both men) and were related to the unscheduled use of glucocorticoids related to severe optic neuropathy followed by orbital radiotherapy.

### 3.2 Primary Outcomes

#### 3.2.1 Changes in Clinical Activity Score

In all patients with baseline CAS 4 and more, CAS has reduced at the end of the observational period, with a median improvement of 4 points at 48 weeks. Disease inactivation (CAS = 0) at the end of the observational period occurred in three patients. Two of them received unscheduled doses of glucocorticoids with orbital radiotherapy.

#### 3.2.2 Proptosis and Diplopia Response

Proptosis was initially observed in 10 eyes. Mean proptosis before treatment was 23.8 mm (max 30 mm), and after the treatment was 21.4 mm. In eight eyes, the reduction of proptosis was equal to or more than 2 mm.

According to Gorman’s score at the initial examination, constant diplopia was present in primary position in four patients, inconstant in one patient, intermittent in one patient, and was absent in one patient. After the treatment with rATG, an improvement of 1 point in Gorman’s score was noticed in three of 6 patients (**Table 2**).

**Table 2:** Diplopia according to the Gorman’s score in patients with active moderate-to-severe GO at baseline and after therapy in patients treated with rATG.

Diplopia	*N* (%)	
Present at baseline	6 (86%)	
Improved at 48 weeks	3 (43%)	
Gorman’s score (patient’s No.)	At baseline	At 48 weeks
1	Constant	Inconstant
2	Constant	Constant
3	Intermittent	Absent
4	Constant	Constant
5	Absent	Absent
6	Constant	Inconstant
7	Inconstant	Inconstant

#### 3.2.3 Best-Corrected Visual Acuity and Mean Retinal Sensitivity

**Figure 1:**
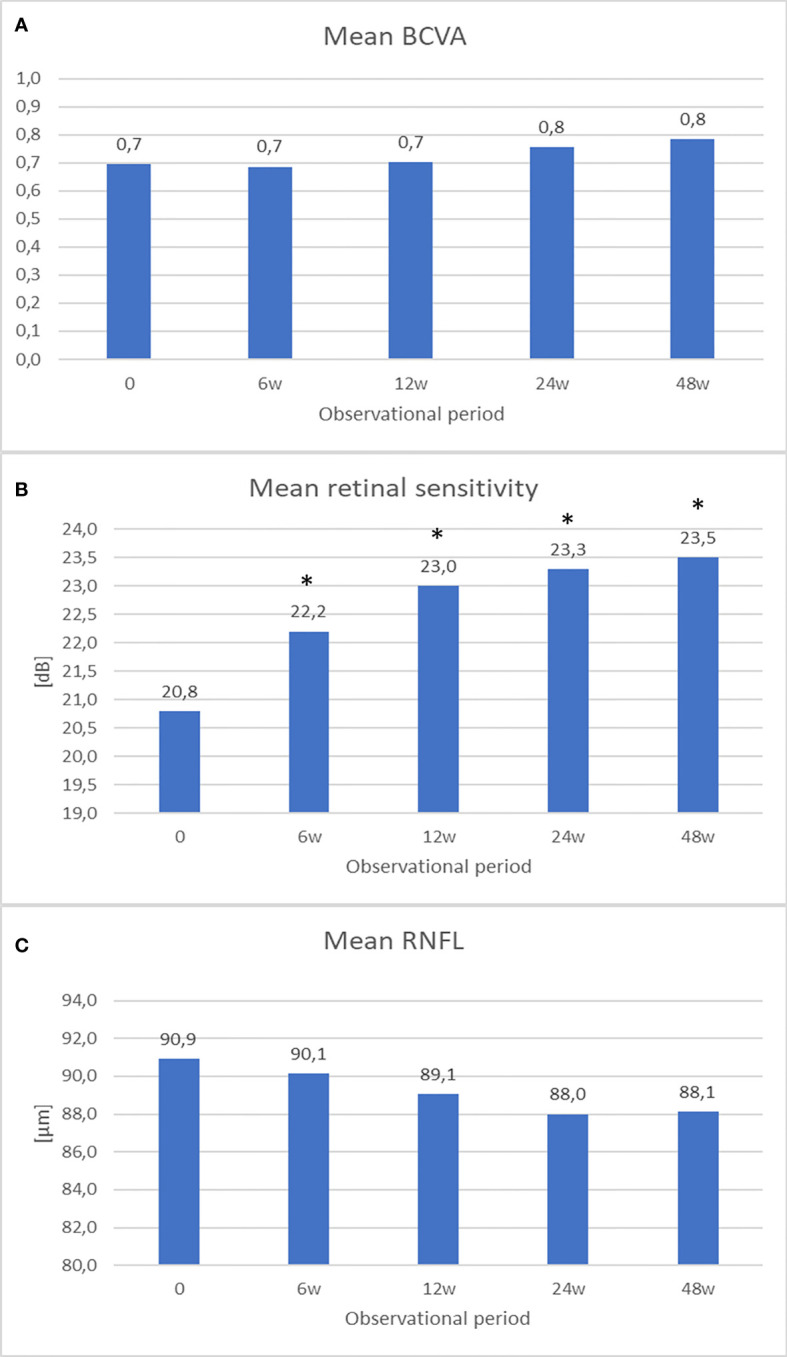
The graphs show **(A)** mean best-corrected visual acuity (BCVA), **(B)** mean retinal sensitivity, **(C)** mean retinal nerve fiber layer (RNFL) – at baseline and at 6, 12, 24, 48 weeks after treatment. *P < 0.05 vs. baseline measurement.

Visual acuity was impaired in 11 eyes. After rATG therapy, the improvement in best-corrected visual acuity (BCVA) was observed in seven eyes (64%) while remaining stable in six eyes. The mean BCVA increased from 0.69 to 0.78 (**Figure 1**). A more significant improvement was noted for eyes with initially worse BCVA in comparison with those with better BCVA before starting rATG treatment.

In one patient with severe optic neuropathy and unscheduled doses of glucocorticoid followed by orbital radiotherapy, the initial BCVA examination showed no light perception in the left eye in the initial period, but, at the end of the study, BCVA has improved to 1.0.

Mean retinal sensitivity (MRS) in the static visual field has improved from 20.8 to 23.5 dB before and at the end of the treatment, respectively (**Figure 1**). The improvement reaches statistical significance at 6-week and all subsequent measurements (*p* < 0.05).

### 3.3 Secondary Outcomes

#### 3.3.1 Optical Coherent Tomography and Retinal Nerve Fiber Layer

In OCT, GCC values remained at a similar level after the treatment, suggesting no further progression of optic neuropathy and further reduction of GCC due to GO.

RNFL thickness slightly decreased in comparison with the initial values after the treatment from 91 to 88 μm (**Figure 1**). In three patients, RNFL decreased ≥2 μm.

#### 3.3.2 Visual Evoked Potentials

The amplitude in 1° tended to increase from 5.86 ± 3.26 to 6.52 ± 1.98 µV, and latencies have elongated in 1°, after the treatment with rATG (**Table 3**).

**Table 3:** Visual evoked potentials.

	Initially	After 48 weeks	*p*-value
**1°**			
Latency (ms)	117 ± 15	119 ± 11	0.78
Amplitude (µV)	5.9 ± 3.3	6.5 ± 2.0	0.37
**15 min**			
Latency (ms)	128 ± 11	137 ± 11	0.05
Amplitude (µV)	8.0 ± 5.5	7.5 ± 4.2	0.52

p-values were calculated with Student’s t-test.

#### 3.3.3 Color Vision

There was a single patient with severe optic neuropathy manifested by severe deterioration of color vision (Ishihara Color Plates RE 11/16, LE 10/16) with full recovery (Ishihara Color Plates RE and LE 16/16) after 24 weeks since the rATG infusion. The patient did not receive unscheduled doses of glucocorticoids/orbital radiotherapy.

#### 3.3.4 Immune Parameters

Five of 7 patients had initially detectable TRAb. One patient became seronegative at the end of the follow-up. One patient obtained about a 40% reduction of TRAb titter after 48 weeks. In the remaining 4 patients, a transient decrease in TRAb values, most prominent at 6 weeks was observed. GO inactivation (assessed by CAS) at the end of follow-up was observed in two subjects with a 40% decline in TRAb titter. In the third patient with the disease inactivation, there was no decline in TRAb titter.

Notwithstanding, a long-lasting improvement in the CD4/CD8 ratio was found in 6 of 7 studied patients. Six of the 7 subjects achieved a 50% reduction of the CD4/CD8 ratio at 6 weeks, which persisted at 48 weeks in 3 of them.

#### 3.3.5 Safety and Adverse Events

No anaphylaxis and local reactions during and after the administration of rATG were noted. There was, however, one case of mild influenza infection (H1N1) within 1 week after rATG administration. In addition, one patient developed serum sickness with fever, rash, myalgia, and pruritus 14 days after treatment. Both patients made a complete recovery.

## 4 Discussion

According to the literature, our study is the first of its kind to assess the usefulness of rATG treatment in steroid-resistant GO in a group of seven patients. Until now, two cases of the patient treated with rATG showing its usefulness in GO were reported (9). The case of GO published by Lee et al. was not previously treated with steroids and received rATG as a part of induction therapy after kidney transplantation. The second one was a part of this study (14). In our series of cases, improvement in the CAS scale was followed by beneficial changes in functional and structural parameters, regardless of the long-lasting activity of moderate-to-severe GO refractory to intravenously administered glucocorticoids.

We have observed long-lasting improvement in CAS and even inactivation of the disease in 3 of 7 patients (43%), reduction of proptosis in 8 eyes (57%), and diplopia in 3 of 6 patients (50%) with the advanced stage of the disease. Of note, an absence of the change in diplopia score in two patients with persistent disease activity may be related to advanced irreversible fibrosis of the extraocular muscles.

In addition, we observed improvement in optic nerve dysfunction probably related to some extent to the reversion of compressive neuropathy after rATG treatment. Advanced damage to the optic nerve and severe fibrosis of extraocular muscles and other intraorbital structures during long-lasting GO caused to a certain degree their irreversible dysfunction.

Visual acuity improved in seven out of 11 eyes, including a spectacular sight recovery in one patient with no light perception to full visual acuity at the end of the study. This proves that visual recovery is possible even in such severely affected patients when optic nerve compression does not cause irreversible damage to the optic nerve fibers.

Mean retinal sensitivity is an important indicator for the evaluation of visual capacity and is frequently used in the measurement of the outcome of as well as pharmacological treatment (15) and surgical decompression for GO (16). In the presented study, increased retinal mean sensitivity at 6-week and all subsequent measurements suggests an improvement of visual function due to rATG treatment.

Dave et al. have shown increased RNFL thickness in the active stage of GO in comparison with the inactive phase (10). Initial increased RNFL thickness in some patients could have been caused by axonal swelling and then resolved with time. As a consequence, the resultant ischemia would have caused a loss of integrity and, therefore, RNFL thinning. These results suggest that patients with more severe Graves’ orbitopathy may have thicker RNFL than patients with a less severe form of the disease in the active phase.

PVEP is a useful method in assessing the function of the optic nerve in GO (17, 18). In our study, an increase in amplitude in 1° PVEP after the treatment with rATG may indicate a decrease of optic nerve compression in GO. Patients included in the study had prolonged optic nerve compression due to steroid-resistant disease, so to some extent, irreversible damage to the optic nerve fibers has occurred. This could be the reason why other parameters, such as latency in 1° PVEP and both latency and amplitude in 15-min PVEP have not improved after rATG treatment. Therefore, PVEP can be used to assess the severity of nerve damage and to monitor dysthyroid optic neuropathy before and after the treatment with rATG.

We have to acknowledge the use of unscheduled doses of glucocorticoids and orbital radiotherapy in two cases, both men, that obtained inactivation of the disease at the end of the study. This observation indicates the potential usefulness of combined therapies in the management of moderate-to-severe GO refractory to intravenously administered glucocorticoids.

The limitation of our study is the small number of participants. Due to the outbreak of the COVID-19 pandemic, we encountered problems associated with low patient attendance, as well as our and the patients’ fear of undergoing immunosuppressive treatment, which could potentially endanger their lives. Despite severe influenza in one patient, no other serious infectious adverse events were present in other patients. Furthermore, viral infection was present in typical winter to early springtime, so to prevent this complication, if possible, rATG should be administered in the summer to early autumn period.

## 5 Conclusions

rATG therapy offers long-lasting improvement in moderate-to-severe steroid-resistant Graves’ orbitopathy with improvement in functional vision (reduction of diplopia, improvement of visual acuity, retinal sensitivity, and VEP pattern). The therapy is well tolerated. However, further research in this field is needed to confirm the usefulness of rATG in the clinical setting in patients with GO.

## Data Availability Statement

The raw data supporting the conclusions of this article will be made available by the authors, without undue reservation.

## Ethics Statement

The studies involving human participants were reviewed and approved by the ethics committee of the Medical University of Silesia. The patients/participants provided their written informed consent to participate in this study.

## Author Contributions

JC and MŚ conceived the concept of the study and designed the study. MŚ, GK, IG-M I, JF, and GH managed the patients. MS-K performed all ophthalmology assessments. KJ performed flow cytometry assessments. MS-K, MŚ, and GH drafted the manuscript. All authors contributed to data interpretation and the writing and editing of the manuscript. JC and EM-K approved the final version of the manuscript. All authors listed have made a substantial, direct, and intellectual contribution to the work and approved it for publication.

## Funding

This research received financial support from the Medical University of Silesia (KNW-1-120/K/9/K).

## Conflict of Interest

The authors declare that the research was conducted in the absence of any commercial or financial relationships that could be construed as a potential conflict of interest.

## Publisher’s Note

All claims expressed in this article are solely those of the authors and do not necessarily represent those of their affiliated organizations, or those of the publisher, the editors and the reviewers. Any product that may be evaluated in this article, or claim that may be made by its manufacturer, is not guaranteed or endorsed by the publisher.
